# Nursing Students’ Experiences About Clinical Practice Tutoring: A Cross-Sectional Observational Study

**DOI:** 10.3390/nursrep14040292

**Published:** 2024-12-13

**Authors:** Álvaro Borrallo-Riego, Eleonora Magni, José Miguel Pérez-Jiménez, María Dolores Guerra-Martín

**Affiliations:** 1 Nursing Department, Faculty of Nursing, Physiotherapy and Podiatry, University of Seville, 41009 Seville, Spain; aborrallo@us.es (Á.B.-R.); jpjimenez@us.es (J.M.P.-J.); guema@us.es (M.D.G.-M.); 2 Institute of Biomedicine of Seville (IBiS), Antonio Maura Montaner Street, 41013 Seville, Spain; 3 Universitary Hospital Virgen Macarena, University of Seville, 41009 Seville, Spain

**Keywords:** nursing students, opinions, clinical practices, clinical tutoring

## Abstract

**Objective:** Nursing students’ experiences about clinical practice tutoring in a public university from southern Spain and at its two attached educational centres were analysed. Methods: A cross-sectional observational study was carried out. The population was comprised of nursing students attending their fourth year of university during the 2023/2024 academic period, with a final sample of 179 subjects. Sociodemographic data were collected and a validated questionnaire on opinions about the figure of clinical practice tutors was applied, with 34 closed questions offering five answer options. The questions were categorized into 17 dimensions. A question on the students’ satisfaction with tutoring of the practices was included, in addition to an open question to gather the students’ comments and/or suggestions in relation to clinical practice tutoring. The analysis was performed through a data matrix in SPSS. The Kruskal–Wallis test was used to compare the centres according to the questionnaire dimensions, considering *p*-values < 0.05 to establish statistically significant differences. The open question was analysed using Atlas.ti. Results: Most of the students were women, with an approximate mean age of 23 years old. Significant differences were detected among the centres in almost all dimensions from the questionnaire. High satisfaction levels were obtained in the three centres. A total of 83 verbatims were collected in the open question and grouped into eight categories. Conclusion: Nursing students place significant importance on the role of the clinical tutor. Clinical tutors who demonstrate enthusiasm for teaching, foster a positive environment based on active listening, respect, and the encouragement of autonomy, are essential to enhancing the students’ experience during clinical placements.

## 1. Introduction

During university training in nursing, clinical practices constitute one of the fundamental pillars for students to acquire knowledge, skills, and competences for their personal and professional development [1,2]. Clinical practices are defined as those activities included in the training process that are carried out by the students and monitored by the universities so that the former can apply and understand the academic knowledge. It will be possible to implement these practices in structures inherent to the university or in collaborating with external entities by means of agreements. For such purpose, the students should have an academic tutor assigned in the university institution and a clinical one in the collaborating entity [2,3,4,5,6]. These assignments are fundamental, as it is necessary that the students’ practical training is properly structured, planned, guided, and evaluated to attain an optimal teaching–learning process [7,8]. In addition, it is necessary for the clinical tutors working in the collaborating entities to provide a permissive and safe environment that also fosters the students’ integration as part of the work team in order to favour the acquisition of the competencies that are required from graduates [9,10].

To assess the students’ experiences in their clinical practices, it becomes necessary to pay attention to the figure of clinical tutors, who are defined as highly experienced professionals who offer support and promote learning and professional development in the students assigned to them [3,11,12]. In addition, various studies point out some characteristics of good clinical tutors, such as adequate emotional stability, maturity, empathy, conflict mediation, communication skills and psychopedagogical training; in addition, they should offer frequent and regular contact with their tutees [13,14]. Nevertheless, the figure of clinical tutors has gradually evolved and adapted to the social context and reality, observing environments with in-person clinical tutors and others with online tutors. This has supposed a change, not only regarding ease of accessing clinical tutors, but also in terms of tutor–student interactions and communication during clinical practices [9,15].

Various studies indicate that the role of a clinical tutor is not sufficiently defined, which is the reason why they may find difficulties knowing their functions and their tutees’ training needs and requirements [3,16]. In fact, many clinical tutors assume the role without adequate preparation or pedagogical guidance, which can result in frustrating experiences for students [17]. For this reason, the training of clinical tutors should be encouraged to ensure that they possess the skills to carry out their role effectively [18]. Given this, various authors speak about the suitability of creating a clinical tutoring system inspired by proper tutor–student communication, where each actor’s functions and responsibilities are clearly defined and the objectives they seek to achieve during the clinical practices are specified [5,19,20,21]. In this way, clinical tutoring becomes highly relevant, as it supports the correct planning and supervision of the practical experience [22].

Along with the figure of clinical tutors, there are other elements that can also affect the tutoring process during clinical practices, namely the students’ motivation to learn; the learning environment; assistance-related overload in the practice service; and interpersonal and communication skills, both in clinical tutors and in students, among others [9,23,24]. In this sense, it is worth mentioning that the clinical practice tutoring experience is a collective responsibility that involves students and clinical tutors alike, with a need for the active involvement of both parties. This aspect will enable the creation of learning environments that foster clinical reflection, autonomy and self-learning in tutees [11,25].

Given the importance of clinical practices and of clinical tutors in nursing studies, it becomes necessary to foster an analysis of the students’ experiences during their practices [26,27,28]. Given all this, the study objective was to analyse nursing students’ experiences in clinical practice tutoring in a public university in southern Spain and at its two attached educational centres.

## 2. Materials and Methods

### 2.1. Design and Participants

**Figure 1:**
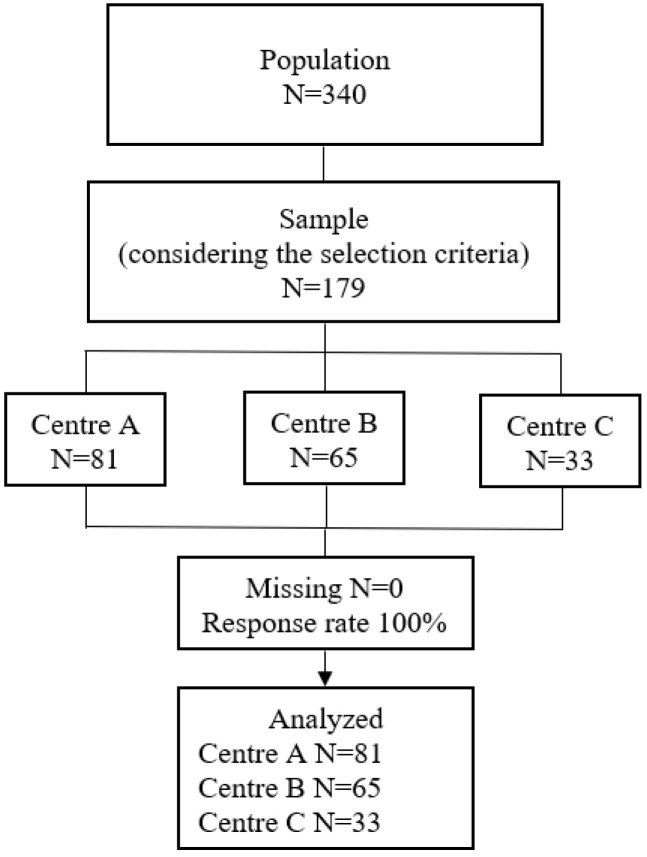
Participant flow chart.

A cross-sectional observational study was carried out [29]. The study population was comprised of 340 students enrolled during the 2023–2024 academic period in the fourth year of the undergraduate Nursing course at a university from southern Spain, which has its own centre and two attached ones, all of which are public institutions and have the same curriculum. Stratified probability sampling was implemented. The following was taken into account for sample calculation: error probability of *p* < 0.05; standardized distance (Z) = 1.96; precision error of 0.05; and population variance of 0.5 for finite populations [29]. Eventually, the sample was comprised of 179 students (see Figure 1).

The following selection criteria were considered to take part in the study. Inclusion criteria: 1. Being undergraduate nursing students attending the fourth year of the course at the university institution or its attached centres where the study was conducted. 2. Voluntarily taking part in the study, with prior signature of an informed consent form. Exclusion criteria: 1. Not having attended any academic discipline from the practicum (in other words, not having undergone clinical practices). 2. Being Erasmus students or of any other type of mobility and/or interchange program.

### 2.2. Study Variables

The following sociodemographic variables were collected: age, gender and educational centre. A questionnaire on opinions about the figure of tutors in clinical practices was used, validated and adapted for the Spanish context by Borrallo-Riego et al. [5], with a Cronbach’s alpha of 0.96. This value reflects excellent internal consistency [30]. The questionnaire was adapted as per the paper by Palacios and Quiroga [31]. It consisted of 34 closed questions focused on the figure of tutors in clinical practices, grouped into 17 dimensions. These questions had five answer options on a Likert scale, from 1. Never to 5. Always. In addition, a closed question about the students’ satisfaction with tutoring was included, also with five answer options from 1. Very dissatisfied to 5. Very satisfied (Table S1). An open question was included at the end of the questionnaire for the students to offer comments and/or suggestions about clinical practice tutoring.

### 2.3. Data Collection

Data collection was carried out between December 2023 and January 2024 during teaching hours in the classrooms at each of the three educational centres included in the study. For such purpose, prior authorisation to enter the classrooms was requested from the principals and faculty of each centre. Once inside the classroom, the students were informed about the study objective, both verbally and in writing. In addition, voluntary participation and signature of an informed consent form were requested. Subsequently, they were presented with the questionnaire, which was self-applied in paper format. Filling in the questionnaire lasted approximately 15 min.

### 2.4. Data Analysis

The analysis of the quantitative variables was performed on a data matrix in the SPSS statistical package for Windows (v.26., IBM Corp., Armonk, NY, USA). A descriptive analysis was performed, indicating frequencies and percentages for each of the questions by centre. Centralisation and dispersion measures were taken for all 18 questionnaire dimensions. The Kruskal–Wallis test was used to contrast the questionnaire dimensions by study centre. Non-parametric statistical tests were employed after applying Kolmogorov–Smirnov and finding out that the data sample did not follow a normal distribution. Additionally, *p*-values < 0.05 were considered to establish statistically significant differences. In the case of the open question included at the end of the questionnaire, Atlas.ti V.22 was used to perform a qualitative and category analysis of the verbatim data. For greater ease in the analysis, the entire process was jointly carried out by two evaluators who worked independently [29]. The verbatims were grouped into eight categories, namely: 1. Primary Health Care/In-hospital Care variability, 2. Treatment given to the students, 3. Willingness to train the students, 4. Interest in tutoring the students, 5. Evaluating the students, 6. Evaluating the clinical tutor, 7. Students’ autonomy, and 8. Work environment.

### 2.5. Ethical Considerations

The study ensured anonymity and confidentiality at all times, taking into account the Declaration of Helsinki and Organic Law No. 3/2018 for Personal Data Protection and Guarantee of Digital Rights, dated December 5th. Prior to taking part in the study, all the students were informed (both verbally and in writing) about the research objectives, asking them to sign an informed consent form. They were informed that participation was voluntary and that they could withdraw their informed consent at any moment. The current study was duly approved by the Andalusian Biomedical Research Ethical Committee (Date: 29 September 2023. Internal Code: 1123-N-23).

## 3. Results

### 3.1. Sociodemographic Characteristics of the Students

The study participants were 179 students with a mean age of 22.96 years old (SD = 3.61), with 86.03% of women (N = 154). The centre-stratified data from the sample are shown in Table 1.

### 3.2. Opinions About the Figure of Tutors in Clinical Practices

Table 2 shows the results obtained in each of the questions included in the questionnaire by centre. In Centre A, questions 1 and 2 were the ones that reached the highest scores. The students from Centre A asserted that the clinical tutors are usually always or almost always punctual and available at the scheduled times. Likewise, they stated that they are usually always or almost always organised in terms of their activities. In Centre B, questions 1 and 14 obtained the highest score. The students from Centre B highlighted the clinical tutors’ punctuality and availability, as well as their ability to encourage the students’ autonomy, safeguarding both the students’ and the patients’ safety. In Centre C, the questions reaching the highest score were 3, 4 and 31. The students from Centre C stated that the clinical tutors always or almost always create positive learning environments, acting as role models thanks to their professional competencies and actively listening to their students. In all three centres, the lowest scores coincided with the same question: number 16. Students from all three centres reported that clinical tutors never or almost never question their tutees’ judgments or capabilities in front of patients.

Table 3 shows the mean scores according to the students’ answers to the questions included in each dimension. The median and interquartile range values are also described, as well as the results after applying the Kruskal–Wallis test for contrasting the centres. As for the mean scores, Dimension 1 (Organisation) was the one obtaining the highest value both in Centre A and in Centre B. However, in Centre C, the highest mean value corresponded to Dimension 3 (Professionalism). Dimension 8 (Respect towards the students) obtained the lowest mean score in all three centres.

The following results were obtained when comparing the findings in each centre according to the questionnaire dimensions: on the one hand, Centre C achieved the highest mean range in all the dimensions. On the other hand, significant differences were detected: (a) between Centres A and B in five dimensions (6, 10, 12, 13 and 15), with better scores in Centre A in all cases; (b) between Centres A and C in three dimensions (5, 8 and 16), with Centre C obtaining better scores in all cases; (c) statistically significant differences were detected between Centres B and C in all the dimensions, except for 4 and 15, with Centre C reaching better results in all of them. Dimensions 14 and 15 were the only ones in which no significant differences were found across all three centres.

### 3.3. Students’ Satisfaction About Clinical Practice Tutoring

Table 4 shows the results related to the students’ satisfaction levels regarding clinical practice tutoring according to each centre. The students from Centre C were the ones stating the highest satisfaction degree, with 94% of them classified as quite or very much satisfied. This was followed by Centre A with 82.8% and, finally, by Centre B with 76.9%. Centre C was the only one where no students stated being little or very little satisfied with tutoring during their clinical practices.

The following results were obtained when comparing the results in question 35 related to satisfaction: on the one hand, Centres C and B reached the highest and lowest mean ranges, respectively. And, on the other hand, no statistically significant differences were detected across the centres.

### 3.4. Students’ Comments or Suggestions in Relation to Clinical Practice Tutoring

Of the entire sample, 68 students (37.99%) answered the open question related to clinical practice tutoring. Additionally, 13 of the students (7.26%) contributed more than one verbatim. There were a total of 83 verbatims. By centre, the students from Centre A were the ones contributing the most verbatims with 50, which represents 60.24% of the total. Centre B ranked second with 19 verbatims (22.89%), followed by Centre C with 14 (16.87%).

In addition, 46.99% of the verbatims (n = 39) were positive. By centre, Centre A reported a total of 20 positive verbatims, followed by Centre B with 11 and Centre C with 8. As for the negative verbatims, they accounted for 53.01% (*n* = 44). By centre, Centre A reported a total of 30 negative verbatims, followed by Centre B with 8 and Centre C with 6.

The verbatims were grouped into eight categories. In Category 1 (Primary Health Care/In-hospital Care variability), the students stated that they usually find differences in the assistance provided by the clinical tutors based on whether the practices are carried out in Primary Health Care or in In-hospital Care settings. Some students stated that clinical tutors are usually more receptive and willing to teach their tutees in a hospital setting. Nevertheless, other opposing verbatims were also described, stating that it is in Primary Health Care that they have found more willingness to teach and pay attention to the students when compared to In-hospital Care. All the verbatims from this category were obtained in Centre A.

In Category 2 (Treatment given to the students), there were positive verbatims stating that the clinical tutors had treated them fine, with respect, closeness and good manners during their clinical practices, providing them with an enjoyable and motivating experience. Nevertheless, some negative verbatims were also expressed, stating that the tutors should improve the way they treat the students and that they should be more empathetic and flexible and enhance their communication skills. Some students noticed neglect and even contempt in how they were treated. Positive and negative verbatims were obtained in all three centres, with similar percentages (54.5% positive against 45.45% negative).

In Category 3 (Willingness to train students), there were positive verbatims indicating that clinical tutors are usually professionals committed to training their tutees and show interest in the teaching–learning process. However, negative verbatims were also expressed, stating that some clinical tutors fail to assist in students’ training, showing little or no interest in their tutees’ learning. Positive and negative verbatims were obtained in all three centres, but the positive ones were more frequent (65.38%). In turn, it was the category receiving the most verbatims from the students.

As for Category 4 (Interest in tutoring the students), only negative verbatims were reported in the three centres. In all the centres, they insisted on the importance of selecting professionals wishing to guide and tutor the students.

In Category 5 (Evaluating the students), only negative verbatims were obtained in all three centres. In this sense, the students stated that they did not agree with the evaluation systems applied in some situations, where grades are assigned with no objectivity or justification. Likewise, they describe that they are not always informed about the characteristics of the evaluation process.

Category 6 (Evaluating the clinical tutor) only received one negative verbatim from a Centre A student. He stated the suitability of incorporating an evaluation of the clinical tutor once the practice period was over. In turn, he proposed devising a protocol for the students to express their complaints regarding their experience with the clinical tutor.

The following findings were obtained in Category 7 (Students’ autonomy): on the one hand, contributions in this aspect were only made in Centres A and C. All the verbatims from Centre A were positive, stating that the clinical tutors grant them independence to perform their tasks. Only one negative verbatim was obtained in Centre C, indicating that some clinical tutors do not allow their students to perform any activity.

Only two verbatims were collected in Category 8 (Work environment). One of them corresponded to Centre A (with negative connotations), stating that some clinical tutors create extremely competitive practice environments. The other verbatim was reported in Centre B—positive in this case. The students stated that the clinical tutors generally create good work environments, rendering a really enjoyable experience.

Table 5 shows some examples of the students’ verbatims in each of the categories described above.

## 4. Discussion

As for the study population sociodemographic characteristics, a high percentage of women with an approximate mean age of 23 years old was recorded. These characteristics coincide with other related studies [5,7,11,32].

In relation to the opinions about the figure of clinical practice tutors, their punctuality and availability during the practice period were highlighted in most of the centres. These findings are in line with various previous studies, which point out that the clinical tutors’ accessibility, willingness and availability are fundamental to ensure proper guidance to the tutees [10,33,34]. In Centre C, the importance of the clinical tutors’ good organisation to foster guidance to the students stood out. In this sense, other authors state that both organisation and planning of the clinical practices should be carried out jointly between students and clinical tutors in order to improve the learning process, ensure a successful experience and increase the satisfaction levels [20,21].

Another aspect that was highlighted was the clinical tutors’ ability to promote the students’ autonomy during the practice period, safeguarding both the students’ and the patients’ safety at all times. Some authors state that certain autonomy degrees should be promoted in the students during their practices, which is fundamental for them to assume responsibility for their own learning, not to mention that it fosters their independence and critical thinking at the same time [9]. To some extent, the autonomy degree depends on the work environment that is generated during the practices. A positive work environment will foster a more permissive and autonomous space for the students, which will improve their learning experience [10,33].

In the opinions, it was also emphasised that clinical tutors should be role models at all times, showing their professional competencies. These findings are in line with various studies which point out that clinical tutors should be role models for their students, showing them skills, knowledge, attitudes and values that inspire them during their training period [13,35,36,37]. In fact, seeing a clinical tutor implementing inadequate learning environments can exert negative effects on the students’ experience during their practices [34].

Another opinion that stood out was related to the importance of promoting communication skills in clinical tutors, who should foster active listening among their tutees. This coincides with the postulates proposed by various authors, who assert that communication skills should be taken care of and attended to in students and clinical tutors alike, as they are fundamental to helping and improving the tutor–student relationship [10,14,38,39].

As for the students’ satisfaction level with clinical practice tutoring, high scores were obtained in all the centres included in the current study. The satisfaction levels obtained in Centres A and C are higher than those described in other studies [11,40,41]. In Centre B, the results are slightly lower than those described by other authors [5].

According to the students’ comments and/or suggestions in relation to clinical practice tutoring, the following considerations were proposed: 1. Reducing variability between Primary Health Care and In-hospital Care, the reason why being it is necessary to pay attention to and consider assistance-related organisation. This aspect has been considered by other authors [42]. 2. Promoting proper treatment and relationships with the students. This coincides with various authors who describe the importance of fostering adequate student–tutor relationships, which should be based on trust and mutual respect [10,43]. 3. Considering the clinical tutors’ willingness to train and mentor the students. Various studies evidence that there should be mutual interest and implication between tutors and students during the entire teaching-learning process [24,33,35]. 4. Reassessing evaluation systems in clinical practices. In this sense, various authors state the need to design suitable evaluation systems that reduce variability in the criteria applied [44,45]. 5. Encouraging positive work environments that foster the students’ autonomy in the practices. These findings are in line with various previous studies [9,10,33,39].

### Limitations

We should mention the research design among the study limitations. Nevertheless, the descriptive observational approach has enabled us to come closer to the students’ reality in terms of clinical practice tutoring. In addition, it has allowed us to notice that there are differences across the different centres and in the characteristics and satisfaction levels at each of them. The fact that data collection has consisted of applying a questionnaire may imply some biases; however, an attempt was made to follow an exhaustive and detailed process in the design to minimize these risks. In fact, one of the most important limitations linked to the questionnaire is that most of its questions are closed, which may have implied loss of exhaustiveness or exclusion of information of interest. An attempt to mitigate this consisted of including the open question as a way to provide the students with a space to freely offer comments and/or suggestions in relation to the topic of our study. Another limitation of the study is that no analysis of differences in the sex variable was performed because more than 86% of the sample were women.

## 5. Conclusions

There was a higher percentage of female students with a similar mean age in all three centres. In general, the nursing students state that the clinical tutors are organised in their work, punctual and available at the scheduled times. Tutors act as role models, showing their competencies in order to foster the students’ professional development. Therefore, it becomes indispensable to create a positive learning environment based on respect and active listening by both parties: tutors and students. This environment should promote the students’ autonomous work, which is crucial to attain independence and encourage critical thinking.

Significant differences in almost all the questionnaire dimensions were detected among the centres. Centre C was the one that obtained the highest average range in all the dimensions. It was not possible to identify significant differences only in two dimensions (14. Intellectual challenge and 15. Fostering interest in the course and its content).

The overall satisfaction level with clinical practice tutoring was high in all three study centres, not detecting significant differences in this sense.

A total of 83 verbatims were reported, most of them in Centre A. Both positive and negative verbatims were reported. The verbatims were grouped into eight dimensions, with the following ones including the most verbatims: Treatment given to the students and Willingness to train the students.

Carrying out an adequate tutoring process during clinical practice has various implications for the teaching–learning process of nursing students. As described, having good tutors who are eager to teach and act as role models significantly enhances the clinical practice experience. Proper tutoring during placements enables students to understand what is expected of them throughout the training process, the objectives they are expected to achieve, and even the assessment process, aspects that are crucial for the student and have a direct impact on their teaching–learning experience.

As a future perspective, the qualitative approach could be expanded with students in order to delve deeper into their experiences during clinical practice. Likewise, the study could be expanded to understand the perspective of clinical tutors in relation to tutoring of practices. In this way, we could understand both perspectives and address suggestions for improvement in greater detail.

## Figures and Tables

**Table 1:** Sociodemographic data of the sample stratified by centre.

Centre	Students N	Women N (%)	Mean Age ± SD
Centre A	81	65 (80.25)	22.39 ± 2.50
Centre B	65	58 (89.23)	22.89 ± 3.03
Centre C	33	31 (93.94)	24.51 ± 5.91

**Table 2:** Results obtained in the questionnaire questions according to the centre.

D & Q		Centre N (%)								
		Centre A			Centre B			Centre C		
		N/AN	S	A/AA	N/AN	S	A/AA	N/AN	S	A/AA
1	Q1	1 (1.2)	9 (11.1)	71 (87.7)	0 (0)	14 (21.5)	51 (78.5)	0 (0)	3 (9.1)	30 (90.1)
	Q2	2 (2.4)	8 (9.9)	71 (87.7)	0 (0)	17 (26.2)	48 (73.8)	0 (0)	3 (9.1)	30 (90.9)
2	Q3	5 (6.2)	13 (16)	63 (77.8)	1 (1.5)	15 (23.1)	49 (75.4)	0 (0)	1 (3)	32 (97)
3	Q4	2 (2.4)	19 (23.5)	60 (74.1)	0 (0)	21 (32.3)	44 (67.7)	0 (0)	1 (3)	32 (97)
	Q5	4 (4.9)	11 (13.6)	66 (81.5)	1 (1.5)	16 (24.7)	48 (73.8)	0 (0)	2 (6)	31 (94)
4	Q6	1 (1.2)	16 (19.8)	64 (79)	0 (0)	19 (29.2)	46 (70.8)	0 (0)	4 (12.1)	29 (87.9)
	Q7	3 (3.7)	13 (16)	65 (80.3)	0 (0)	19 (29.2)	46 (70.8)	0 (0)	2 (6)	31 (94)
5	Q8	8 (9.9)	15 (18.5)	58 (71.6)	3 (4.6)	19 (29.2)	43 (66.2)	0 (0)	2 (6)	31 (94)
	Q9	3 (3.7)	11 (13.6)	67 (82.7)	3 (4.6)	16 (24.7)	46 (70.7)	0 (0)	4 (12.1)	29 (87.9)
	Q10	2 (2.4)	11 (13.6)	68 (84)	1 (1.5)	15 (23.1)	49 (75.4)	0 (0)	4 (12.1)	29 (87.9)
6	Q11	4 (4.9)	16 (19.8)	61 (75.3)	3 (4.6)	15 (23.1)	47 (72.3)	2 (6)	7 (21.2)	24 (72.7)
	Q12	8 (9.9)	10 (12.3)	63 (77.8)	8 (12.3)	19 (29.2)	38 (58.5)	0 (0)	6 (18.2)	27 (81.8)
	Q13	6 (7.4)	18 (22.2)	57 (70.4)	6 (9.2)	18 (27.7)	41 (63.1)	4 (12.1)	5 (15.2)	24 (72.7)
7	Q14	8 (9.9)	13 (16)	60 (74.1)	1 (1.5)	13 (20)	51 (78.5)	0 (0)	3 (9.1)	30 (90.1)
8	Q15	5 (6.2)	7 (8.6)	69 (85.2)	1 (1.5)	14 (21.5)	50 (77)	0 (0)	2 (6)	31 (94)
	Q16	51 (62.9)	11 (13.6)	19 (23.5)	15 (23.1)	23 (35.4)	27 (41.5)	21 (63.6)	5 (15.2)	7 (21.2)
	Q17	14 (17.3)	14 (17.3)	53 (65.4)	6 (9.2)	24 (37)	35 (53.8)	2 (6)	7 (21.2)	24 (72.7)
9	Q18	4 (4.9)	19 (23.5)	58 (71.6)	2 (3)	20 (30.8)	43 (66.2)	0 (0)	3 (9.1)	30 (90.1)
10	Q19	8 (9.9)	8 (9.9)	65 (80.2)	1 (1.5)	23 (35.4)	41 (63.1)	0 (0)	5 (15.2)	28 (84.8)
	Q20	8 (9.9)	9 (11.1)	64 (79)	2 (3)	17 (26.2)	46 (70.8)	3 (9.1)	6 (18.2)	24 (72.7)
11	Q21	8 (9.9)	13 (16)	60 (74.1)	3 (4.6)	22 (33.9)	40 (61.5)	0 (0)	3 (9.1)	30 (90.1)
12	Q22	7 (8.6)	12 (14.8)	62 (76.6)	2 (3)	17 (26.2)	46 (70.8)	0 (0)	6 (18.2)	27 (81.8)
	Q23	6 (7.4)	8 (9.9)	67 (82.7)	2 (3)	21 (32.3)	42 (64.7)	0 (0)	5 (15.2)	28 (84.8)
	Q24	3 (3.7)	10 (12.3)	68 (84)	1 (1.5)	17 (26.2)	47 (72.3)	0 (0)	4 (12.1)	29 (87.9)
13	Q25	1 (1.2)	10 (12.3)	70 (86.5)	2 (3)	17 (26.2)	46 (70.8)	0 (0)	4 (12.1)	29 (87.9)
	Q26	7 (8.6)	11 (13.6)	63 (77.8)	4 (6.1)	20 (30.8)	41 (63.1)	1 (3)	8 (24.3)	24 (72.7)
14	Q27	9 (11.1)	9 (11.1)	63 (77.8)	0 (0)	20 (30.8)	45 (69.2)	1 (3)	3 (9.1)	29 (87.9)
15	Q28	7 (8.6)	17 (21)	57 (70.4)	1 (1.5)	19 (29.2)	45 (69.2)	1 (3)	6 (18.2)	26 (78.8)
16	Q29	8 (9.9)	16 (19.7)	57 (70.4)	2 (3)	21 (32.3)	42 (64.7)	0 (0)	5 (15.2)	28 (84.8)
	Q30	15 (18.5)	27 (33.3)	39 (48.2)	4 (6.1)	27 (41.5)	34 (52.4)	5 (15.2)	9 (27.3)	19 (57.5)
	Q31	6 (7.4)	8 (9.9)	67 (82.7)	1 (1.5)	20 (30.8)	44 (67.7)	0 (0)	1 (3)	32 (97)
17	Q32	7 (8.6)	13 (16.1)	61 (75.3)	1 (1.5)	19 (29.2)	45 (69.2)	2 (6)	3 (9.1)	28 (84.8)
	Q33	6 (7.4)	12 (14.8)	63 (77.8)	1 (1.5)	21 (32.3)	43 (66.2)	2 (6)	5 (15.2)	26 (78.8)
	Q34	8 (9.9)	14 (17.3)	59 (72.8)	2 (3)	19 (29.2)	44 (67.7)	0 (0)	4 (12.1)	29 (87.9)

D & Q: Dimensions and Questions; N/AN: Never/Almost never; S: Sometimes; A/AA: Always/Almost always.

**Table 3:** Results according to the questionnaire dimensions. Kruskal–Wallis contrast test.

Dimensions	Centre	Mean ± SD	Median/IQR	AR	S	*p*	CT	SS
1. Organisation	A	4.37 ± 0.79	5.0/1.0	183.88	12.65	0.0017	A-B	-
	B	4.18 ± 0.79	4.0/1.0	159.28			A-C	-
	C	4.57 ± 0.65	5.0/1.0	208.56			B-C	*
2. Setting	A	4.29 ± 0.99	5.0/1.0	92.52	8.07	0.0176	A-B	-
	B	4.13 ± 0.82	4.0/1.0	78.56			A-C	-
	C	4.63 ± 0.54	5.0/1.0	106.33			B-C	*
3. Professionalism	A	4.19 ± 0.90	4.0/1.0	173.78	21.81	0.0000	A-B	-
	B	4.11 ± 0.85	4.0/2.0	162.14			A-C	*
	C	4.68 ± 0.55	5.0/1.0	227.72			B-C	*
4. Communication skills	A	4.28 ± 0.88	5.0/1.0	181.45	13.01	0.0014	A-B	-
	B	4.13 ± 0.83	4.0/2.0	160.59			A-C	-
	C	4.57 ± 0.65	5.0/1.0	211.93			B-C	*
5. Clarity and comprehensibility	A	4.21 ± 0.92	4.0/1.0	270.23	22.54	0.0000	A-B	-
	B	4.05 ± 0.90	4.0/2.0	239.67			A-C	*
	C	4.55 ± 0.67	5.0/1.0	323.74			B-C	*
6. Feedback to the students	A	4.11 ± 0.98	4.0/2.0	278.97	22.95	0.0000	A-B	*
	B	3.81 ± 1.02	4.0/2.0	232.97			A-C	-
	C	4.30 ± 1.06	5.0/1.0	315.47			B-C	*
7. Students’ autonomy	A	4.11 ± 1.04	4.0/2.0	89.07	7.42	0.0244	A-B	-
	B	4.07 ± 0.81	4.0/1.0	81.34			A-C	-
	C	4.54 ± 0.66	5.0/1.0	109.31			B-C	*
8. Respect towards the students	A	3.49 ± 1.52	4.0/3.0	256.86	17.97	0.0001	A-B	-
	B	3.67 ± 1.07	4.0/2.0	254.95			A-C	*
	C	4.22 ± 0.93	4.0/1.0	326.43			B-C	*
9. Perceived learning achievements	A	4.02 ± 0.97	4.0/2.0	88.95	8.48	0.0143	A-B	-
	B	3.92 ± 0.85	4.0/2.0	80.66			A-C	-
	C	4.45 ± 0.66	5.0/1.0	110.95			B-C	*
10. Equality in the professor’s attitudes	A	4.20 ± 1.07	5.0/1.0	193.63	17.24	0.0001	A-B	*
	B	3.93 ± 0.82	4.0/2.0	151.54			A-C	-
	C	4.31 ± 0.91	5.0/1.0	199.87			B-C	*
11. Enthusiasm for the academic discipline and for teaching	A	4.07 ± 1.06	4.0/2.0	92.17	14.33	0.0007	A-B	-
	B	3.83 ± 0.87	4.0/2.0	75.03			A-C	-
	C	4.54 ± 0.66	5.0/1.0	114.13			B-C	*
12. Availability and willingness to help	A	4.21 ± 0.95	4.0/1.0	284.45	25.31	0.0000	A-B	*
	B	3.95 ± 0.82	4.0/2.0	229.10			A-C	-
	C	4.41 ± 0.74	5.0/1.0	309.64			B-C	*
13. Knowledge about the subject matter	A	4.21 ± 0.91	4.0/1.0	191.67	14.83	0.0005	A-B	*
	B	3.89 ± 0.93	4.0/2.0	153.67			A-C	-
	C	4.30 ± 0.87	5.0/1.0	200.47			B-C	*
14. Intellectual challenge	A	4.17 ± 1.09	5.0/1.0	94.51	7.95	0.0187	A-B	-
	B	4.0 ± 0.79	4.0/2.0	76.69			A-C	-
	C	4.42 ± 0.79	5.0/1.0	102.56			B-C	-
15. Fostering interest in the course and its content	A	4.02 ± 1.09	4.0/2.0	91.18	2.33	0.3118	A-B	-
	B	3.98 ± 0.81	4.0/2.0	83.74			A-C	-
	C	4.24 ± 0.86	4.0/1.0	99.40			B-C	-
16. Fostering discussions and opinions	A	3.88 ± 1.15	4.0/2.0	269.84	19.45	0.0000	A-B	-
	B	3.81 ± 0.83	4.0/1.0	241.23			A-C	*
	C	4.27 ± 0.93	5.0/1.0	321.62			B-C	*
17. Sensitivity regarding the class level and its progress	A	4.13 ± 1.04	4.0/1.0	280.52	25.02	0.0000	A-B	*
	B	3.91 ± 0.82	4.0/2.0	230.89			A-C	-
	C	4.38 ± 0.85	5.0/1.0	315.77			B-C	*

IQR: Interquartile range; AR: Average range; S: Statistical; *p*: *p*-value; CT: Contrast; SS: statistical significance; -: No statistical significance; *: Yes statistical significance.

**Table 4:** Results and comparison of the question regarding students’ satisfaction with clinical practice tutoring.

Question	C	N (%)			Mean ± SD	M/IQR	AR	S	*p*	CT	SS
		VD/NVS	SS	QS/VS							
Q35 (Satisfaction)	A	7 (8.6)	7 (8.6)	67 (82.8)	4.24 ± 1.09	5.0/1.0	95.9	8.01	0.018	A-B	-
	B	3 (4.5)	12 (18.5)	50 (76.9)	4.01 ± 0.92	4.0/1.0	76.8			A-C	-
	C	0 (0)	2 (6)	31 (94)	4.48 ± 0.61	5.0/1.0	101.2			B-C	-

C: Centre; VD: Very dissatisfied; NVS: Not very satisfied; SS: Somewhat satisfied; QS: Quite satisfied; VS: Very satisfied; M: Median; IQR: Interquartile range; AR: Average range; S: Statistical; *p*: *p*-value; CT: Contrast; SS: statistical significance; -: No statistical significance.

**Table 5:** Categorization of the students’ verbatims.

Categories	N	Examples of the Students’ Verbatims
1. Primary Health Care/ In-hospital Care variability	4	*“In the hospitals, clinical tutors are usually more receptive and willing to teach than in Primary Health Care” (S.52/C.A).* *“In Primary Health Care…clinical tutors tend to be more interested in what you learn than in the hospitals” (S.76/C.A).*
2. Treatment given to the students	26	*“I’ve felt motivated from the first moment thanks to how I was treated in the practices. It’s all very close…” (S.33/C.A).* *“He’s always treated me fine. He’s been by my side and I’ve felt supported at all times” (S.130/C.B).* *“He’s always treated me fine and has adapted to my needs” (S.155/C.C).* *“…They should improve communication with the students. Be more assertive and not despise” (S.55/C.A).* *“…They should be more flexible and empathetic to the practicing students” (S.135/C.B).* *“Some tutors have treated me bad and ended up demotivating me” (S.161/C.C).*
3. Willingness to train students	29	*“Almost all the clinical tutors are willing to teach and make teaching really enjoyable” (S.13/C.A).* *“The clinical tutors…have always shown great interest in my learning…” (S.110/C.B).* *“…Most of them are usually willing to teach” (S.173/C.C).* *“…Some tutors don’t collaborate in the students’ training or worry for what we’re learning” (S.11/C.A).* *“…The clinical tutor should keep a better eye on our training and be more willing to teach” (S.95/C.B).* *“…Some tutors don’t even bother to teach. They don’t explain anything” (S.164/C.C)*
4. Interest in tutoring the students	11	*“To ease learning, they should designate as clinical tutors professionals that are really interested in doing so” (S.38/C.A).* *“It’s important that the clinical tutor wants to mentor the students. Some of them don’t want to have students and they’re our main resource if we have any problem” (S.93/C.B).* *“They should look for people that are involved and interested in tutoring the students” (S.156/C.C).*
5. Evaluating the students	6	*“…I’d like to be given more feedback about how they’re going to evaluate me” (S.25/C.A).* *“Sometimes they grade us without even knowing us, with no justification whatsoever” (S.125/C.B).* *“Improving objectivity when grading us” (S.158(C.C).*
6. Evaluating the clinical tutor	1	*“The clinical tutor should be evaluated a posteriori…a protocol should be created to file complaints” (S.53/C.A).*
7. Students’ autonomy	4	*“…They trust me to perform any task” (S.28/C.A).* *“…Some of them don’t let you do anything…” (S.162/C.C).*
8. Work environment	2	*“Some clinical tutors create very competitive environments with our classmates” (S.42/C.A).* *“…The clinical tutor’s created a good work climate…he’s made the environment really enjoyable” (S.127/C.B).*
**Total verbatims**	**83**	

## Data Availability

The original contributions presented in the study are included in the article; further inquiries can be directed to the corresponding author.

## Supplementary Materials

The following supporting information can be downloaded at: https://www.mdpi.com/article/10.3390/nursrep14040292/s1, Table S1: Questionnaire on opinions about the figure of tutors in clinical practices.

## Public Involvement Statement

No public involvement in any aspect of this research.

## Guidelines and Standards Statement

The manuscript was drafted against the Cheklist for reporting results using observational descriptive studies as research designs [46].
