# Supplementary material for: Nursing Students’ Experiences About Clinical Practice Tutoring: A Cross-Sectional Observational Study
Source: Nurs Rep. 2024 Dec 13;14(4):3993–4005. doi: 10.3390/nursrep14040292 (PMC11678620; doi:10.3390/nursrep14040292)
Supplement: Supplementary file 1 [file nursrep-14-00292-s001.zip › nursrep-3367359-supplementary.pdf]

Table S1. Questionnaire on opinions about the figure of tutors in clinical practices.

**Dimensions**	**Questions asked**
1. Organisation	Q1: Is he/she punctual and available at the scheduled times?<br>Q2: Is he/she organized in terms of his/her activities?
2. Setting	Q3: Does he/she create a positive learning environment that is free from tensions, tolerant, quiet and patient?
3. Professionalism	Q4: Is he/she a role model thanks to his/her professional competence?<br>Q5: Does he/she take his/her job as a professor seriously?
4. Communication skills	Q6: Is he/she expressive, both in his/her verbal and non-verbal language?<br>Q7: Is he/she accessible and communicative?
5. Clarity and comprehensibility	Q8: Is he/she clear when communicating the behaviours, roles and performance that he/she expects from the students.<br>Q9: Does he/she explain the concepts and techniques in a clear and summarised way?<br>Q10: Does he/she answer questions in detail?
6. Feedback to the students	Q11: Does he/she analyse the students' work indicating what they do correctly and incorrectly?<br>Q12: Does he/she provide frequent and constructive feedback?<br>Q13: Does he/she explain to the students the reasons why their work is not [illegible]
7. Students' autonomy	Q14: Does he/she allow the students to work as independently as
8. Respect towards the students	Q15: Is he/she respectful and does not make the students feel intimidated or out of place?<br>Q16. Does he/she question the students' capabilities in front of the patients?<br>Q17: Does he/she praise the students in front of patients, professors and/or peers?
9. Perceived learning achievements	Q18: Does he/she teach current and pertinent clinical concepts and procedures?
10. Equality in the professor's attitudes	Q19: Is his/her treatment impartial and does he/she treat the students with no preferences?<br>Q20: Do his/her grades reflect the students' work quality?
11. Enthusiasm for the academic discipline and for teaching	Q21: Does he/she enjoy teaching, showing interest for the students to learn?
12. Availability and willingness to help	Q22: Does he/she work hard and goes beyond his/her assistance-related duties?<br>Q23: Does he/she guide the students by providing tips and suggestions?<br>Q24: Is he/she available and receptive?
13. Knowledge about the subject matter	Q25: Does he/she show knowledge and clinical skills according to his/her specialty area?<br>Q26: Does he/she show procedures on the patients as a role model?
14. Intellectual challenge	Q27: Does he/she motivate the students for them to achieve the best performance possible?
15. Fostering interest in the course and its content	Q28: Does he/she manage to make learning interesting, enjoyable and fun?

16. Fostering discussions and opinions	Q29: Is he/she flexible and open to different points of view?<br>Q30: I the clinical tutor capable of criticising his/her own performance?<br>Q31: Does he/she listen carefully to the students?
17. Sensitivity regarding the class level and its progress	Q32: Does he/she consider the students' knowledge and experience levels when teaching?<br>Q33: Does he/she show interest in the students' difficulties?<br>Q34: Does he/she motivate the students and ask them about their work?
Satisfaction question	Q35: Please rate your overall satisfaction.

Source: [5,27]

References

5 Borrallo-Riego, Á.; Magni, E.; Jiménez-Álvarez, J.A.; Fernández-Rodríguez, V.; Guerra-Martín, M.D. Health Sciences Students' Perceptions of the role of the supervisor in clinical placements. *Int. J. Environ. Res. Public Health* **2021**, *18*, 4427. https://doi.org/10.3390/ijerph18094427.

27 Lima-Rodríguez, J.S.; Lima-Serrano, M.; Ponce-González, J.A.; Guerra-Martin, M.D. Diseño y validación de contenido de rúbricas para evaluar las competencias prácticas en estudiantes de Enfermería. *Educ. Médica Super.* **2015**, *29*, 119–133.
